# Sirolimus Restores Erythropoiesis and Controls Immune Dysregulation in a Child With Cartilage-Hair Hypoplasia: A Case Report

**DOI:** 10.3389/fimmu.2022.893000

**Published:** 2022-05-19

**Authors:** Giovanni Del Borrello, Maurizio Miano, Concetta Micalizzi, Michela Lupia, Isabella Ceccherini, Alice Grossi, Andrea Cavalli, Stefano Gustincich, Marta Rusmini, Maura Faraci, Gianluca Dell’Orso, Ugo Ramenghi, Alessio Mesini, Erica Ricci, Maurizio Schiavone, Natascia Di Iorgi, Carlo Dufour

**Affiliations:** ^1^ Hematology Unit, Istituto di Ricerca e Cura a Carattere Scintifico (IRCCS) Istituto Giannina Gaslini, Genoa, Italy; ^2^ Unitá Operativa Semplice Dipartimentale (UOSD) Genetics and Genomics of Rare Diseases, Istituto di Ricerca e Cura a Carattere Scintifico (IRCCS) Istituto Giannina Gaslini, Genoa, Italy; ^3^ Italian Institute of Technology (IIT), Genoa, Italy; ^4^ Hematopoietic Stem Cell Transplantation Unit, Istituto di Ricerca e Cura a Carattere Scintifico (IRCCS) Istituto Giannina Gaslini, Genoa, Italy; ^5^ Haematology Unit, Regina Margherita Hospital, Turin, Italy; ^6^ Department of Public Health and Pediatrics, School of Medicine, University of Turin, Turin, Italy; ^7^ Covid Hospital, Unità Operativa di Malattie Infettive, Dipartimento di Scienze Pediatriche, Istituto di Ricerca e Cura a Carattere Scintifico (IRCCS) Istituto Giannina Gaslini, Genoa, Italy; ^8^ Pediatric Unit, Santa Maria Annunziata Hospital, Taranto, Italy; ^9^ Department of Neuroscience, Rehabilitation, Ophthalmology, Genetics, Maternal and Child Health, University of Genova, Genoa, Italy; ^10^ Department of Pediatrics, Istituto di Ricerca e Cura a Carattere Scintifico (IRCCS) Istituto Giannina Gaslini, Genoa, Italy

**Keywords:** sirolimus, bone marrow failure disorders, cartilage-hair hypoplasia (CHH), pure red cell aplasia (PRCA), whole-genome sequencing (WGS)

## Abstract

Cartilage-hair hypoplasia (CHH) is a syndromic immunodeficiency characterized by metaphyseal dysplasia, cancer predisposition, and varying degrees of anemia. It may present as severe combined immunodeficiency in infancy, or slowly progress until fully manifesting in late adolescence/adulthood. No targeted treatment is currently available, and patients are usually managed with supportive measures, or are offered a bone marrow transplant if the clinical phenotype is severe and a suitable donor is available. We report the case of a young girl presenting with transfusion-dependent erythropoietic failure and immunological features resembling autoimmune lymphoproliferative syndrome who responded well to empirical sirolimus. She later developed a marked growth delay, which was ultimately attributed to metaphyseal dysplasia. A diagnosis of CHH was reached through whole-genome sequencing (WGS), after a less sensitive genetic diagnostic strategy failed. The patient eventually underwent a haploidentical bone marrow transplant due to progressive combined immunodeficiency manifested as cryptococcal meningoencephalitis. This case illustrates the potential role of sirolimus in correcting anemia and partially controlling the immune aberrations associated with CHH, and serves as a reminder of the invaluable role of WGS in diagnosing patients with complex and atypical presentations.

## Introduction

Cartilage-hair hypoplasia (CHH, OMIM # 250250) is a syndromic immunodeficiency disorder whose cardinal features are metaphyseal dysplasia, fine and sparse hair, Hirschsprung disease or chronic/recurrent non-infectious diarrhea, cancer predisposition, and anemia (1). From a genetic standpoint, CHH is due to recessively inherited mutations in the untranslated RNA component of mitochondrial RNA-processing (*RMRP*) endoribonuclease. Pathogenic mutations in *RMRP* disrupt multiple cellular functions (2, 3), namely, ribosomal processing, cell-cycle progression, telomere maintenance, and epigenetic regulation through small interfering RNAs.

The immunological phenotype of CHH is highly variable, even among siblings with the same genotype, ranging from isolated laboratory abnormalities (i.e., reduced peripheral T-cell proliferation and defective thymic output) to symptomatic hypogammaglobulinemia, to severe combined immunodeficiency and immune dysregulation (4), which may either appear in infancy or slowly progress until fully manifesting in late adolescence/adulthood (5). From a hematological perspective, most CHH patients show a mild macrocytic anemia, which usually starts in early infancy and resolves spontaneously by late childhood (6). Nonetheless, approximately 10% of patients are chronically transfusion-dependent with severe growth impairment of erythroid precursors (7). A small minority of patients display autoimmune hemolytic anemia, usually in the context of widespread immune dysregulation (4).

CHH is currently managed only with supportive treatments (i.e., red blood cell transfusions, immunoglobulin substitution, and antibiotic prophylaxis). Hematopoietic stem cell transplantation (HSCT) does not obviously correct skeletal abnormalities but rescues marrow failure and immune derangement (8), and may thus be offered to patients with transfusion-dependent erythroid failure and clinically apparent combined immunodeficiency.

Here, we report the first successful pharmacologic treatment of CHH-related erythropoietic failure and immune dysregulation with sirolimus.

## Case Report

A 3-year-old girl was referred to our unit for evaluation of congenital transfusion-dependent anemia. She was born at 38 weeks gestational age, small for gestational age (SGA) for length (42 cm), but of normal weight (3,260 g). Before admission, her hemoglobin (Hb) values fluctuated between 5 and 10 g/dl, requiring approximately 1 unit of concentrated red blood cell every 3 to 4 months. Erythropoietin supplementation 500 U/kg/week and prednisone 1 mg/kg/day were not able to improve anemia. On clinical examination, she showed black curly hair, no visible hand or skeletal malformations, or abnormal facial features; splenomegaly was noted and later confirmed by ultrasonography (11 cm in longitudinal diameter, without signs of hepatic cirrhosis or portal hypertension). Upon laboratory evaluation, she showed borderline macrocytosis (mean corpuscular volume 95 fl, range of normal for age 74–94 fl), non-compensatory reticulocyte count (56,000/μl), elevated erythropoietin levels (3,316 IU/L, normal values 3.7–31.5 IU/L), increased Hb F (5.1%, normal values < 1%) and vitamin B12 (1,263 pg/ml, normal values 190–660 pg/ml), normal iron balance, no evidence of hemolysis, and a mildly positive Direct Antiglobulin Test (DAT). Polymerase chain reaction for Parvovirus B19 DNA was negative. Erythrocyte adenosine deaminase (eADA) was increased at 8.4 U/gHb (normal values < 1.2 U/gHb). Bone marrow biopsy revealed erythroid hypoplasia, as reflected by an increased granulocyte/erythroid precursor ratio (4/1), and some degree of dyserythropoiesis. *In vitro* erythroid precursor growth (methyl cellulose assay) was severely impaired compared to normal controls and was not rescued after lymphodepletion of the sample. Patient bone marrow plasma did not inhibit erythroid colony formation from the marrow of a healthy donor. These features were in keeping with the intrinsic erythropoietic failure associated with Diamond Blackfan Anemia (DBA), but both Sanger sequencing and the Multiple Ligand Probe Assay of the genes more frequently associated with DBA (RPS19, RPL5, RPL11, RPS17, RPS26, and RPL35a) did neither reveal any single-nucleotide variants or copy number variants. The dyepoxybutane chromosomal fragility test was normal, thus excluding Fanconi anemia and related genetic DNA repair defects. Telomere length, assayed by flow-fluorescence *in situ* hybridization (FISH), was normal in granulocytes and unmeasurable in lymphocytes due to the insufficient number of cells to perform the test. Ribosomal RNA (rRNA) analysis (i.e., quantification of long non-coding rRNA precursors, which accumulate in the case of ribosomal processing malfunction) (9) was attempted on multiple occasions but failed due to the insufficient quantity of nucleic acid retrieved from the patient’s lymphocytes.

At 7.5 years of age, the patient was hospitalized for septic shock due to *Streptococcus viridans*. At this point in time, she was mildly lymphopenic, with undetectable serum IgA and normal IgG and IgM values. Further immunologic testing revealed low CD4+ and CD8+ T lymphocytes, decreased mitogen-induced lymphocyte proliferation, moderately increased alpha-beta T lymphocyte CD4-CD8- (“double-negative” T lymphocytes, DNTs), and markedly reduced thymic output (i.e., low naive T cell and absent T excision circles) (**Table 1**). FAS-mediated lymphocyte apoptosis was within the normal limit. A neck ultrasound revealed thymic hypoplasia. FISH analysis of chromosome 22 was normal, most likely excluding a diagnosis of Di George syndrome. A custom panel of 312 genes related to primary immune deficiencies and bone marrow failure syndromes was analyzed through a next-generation sequencing (NGS) tool in use in our center (10) but no pathogenic variants were detected. This version of our NGS panel did not evaluate for the RMRP gene, which was included in later versions. Whole-exome sequencing (WES) was then performed, and it did not detect any significant mutation. Prophylaxis with trimethoprim-sulfamethoxazole was started to prevent opportunistic infections due to *Pneumocystis jiroveci*.

**Table 1:** Hematological and immunological features of the patient.

	Age 7.5 years (before sirolimus)	Reference range for age	Age 14.5 years (sirolimus steady state)	Reference range for age
**Height**	115 cm	5th centile for age and sex	142.5 cm	−2.9 SDS for age and sex
**Spleen size**	12 cm	7–9.5 cm	10 cm	9–12 cm
**Hemoglobin**	7.5 g/dl	11–15 g/dl	12	11.5–15.5
**Mean cellular volume**	95 fl	78–94 fl	85 fl	80–98 fl
**Reticulocyte count**	15,000/mmc	20,000–100,000/mmc	50,000/mmc	20,000–100,000/mmc
**Erythroid colony-forming unit count**	3	27–81/2 × 10^4^	40	27–81/2 × 10^4^
**White blood cell count**	6,000/mmc	4,000–10,000/mmc	6,500/mmc	4,000–10,000/mmc
**Lymphocyte count**	1,370/mmc	1,500–6,000/mmc	500/mmc	1,350–4,500/mmc
**T-lymphocyte count**	710/mmc	1,100–4,600/mmc	235/mmc	900–3,000/mmc
**CD4+ T-lymphocyte count**	435/mmc	650–3,500/mmc	200/mmc	550–2,000/mmc
**CD8+ T-lymphocyte count**	95/mmc	300–1,500/mmc	25/mmc	250–1,000/mmc
**B-lymphocyte count**	220/mmc	250–750/mmc	37/mmc	150–650/mmc
**Natural killer count**	380/mmc	120–400/mmc	185/mmc	90–500/mmc
**DNT lymphocyte count**	5% (of total lymphocytes)	<1.7% (of total lymphocytes)	0.5% (of total lymphocytes)	<1.7% (of total lymphocytes)
**B220-positive DNT lymphocyte count**	75% (of DNT lymphocytes)	<55% (of DNT lymphocytes)	0% (of DNT lymphocytes)	<55% (of DNT lymphocytes)
**CD45RA T-lymphocyte count**	21% (of T lymphocytes)	45%–70% (of T lymphocytes)	5% (of T lymphocytes)	35%–65% (of T lymphocytes)
**CD45RO T-lymphocyte count**	77% (of T lymphocytes)	25%–50% (of T lymphocytes)	90% (of T lymphocytes)	35%–65% (of T lymphocytes)
**CD27 B-lymphocyte count**	16% (of B lymphocytes)	>15% (of B lymphocytes)	0%	>15% (of B lymphocytes)
**IgG level**	750 mg/dl	550–1400 mg/dl	820 mg/dl*	700–1,600 mg/dl
**PHA-stimulated T-lymphocyte count**	28 × 10^3^ cpm	215 × 10^3^ cpm	–	–
**TRECs**	<100/ml	>7,770/ml	–	–

Findings clearly show overtime improvement in erythropoiesis and red blood cell parameters, amelioration of immunodysregulation, but progression of immunodeficiency. DNT, double-negative T lymphocytes; PHA, phytohemagglutinin; TREC, T-cell receptor excision circles. *Under subcutaneous immunoglobulin replacement.

Since splenomegaly, increased DNTs, increased B12 serum level, reduced thymic output, and positive DAT were consistent with a diagnosis of the primary immune regulatory disorder (PIRD) autoimmune lymphoproliferative syndrome (ALPS), we started treating our patient with sirolimus (2 mg/mq/day) (11): her Hb values improved, reaching a plateau at 12–13 g/dl, DAT became negative, spleen volume progressively reduced to normal (8 cm of longitudinal diameter after 6 months of therapy), and DNTs decreased below 1%. Trephine biopsy showed normal erythroid cellularity and only mild signs of dyserythropoiesis; *in vitro* erythroid precursor growth became normal.

**Figure 1:**
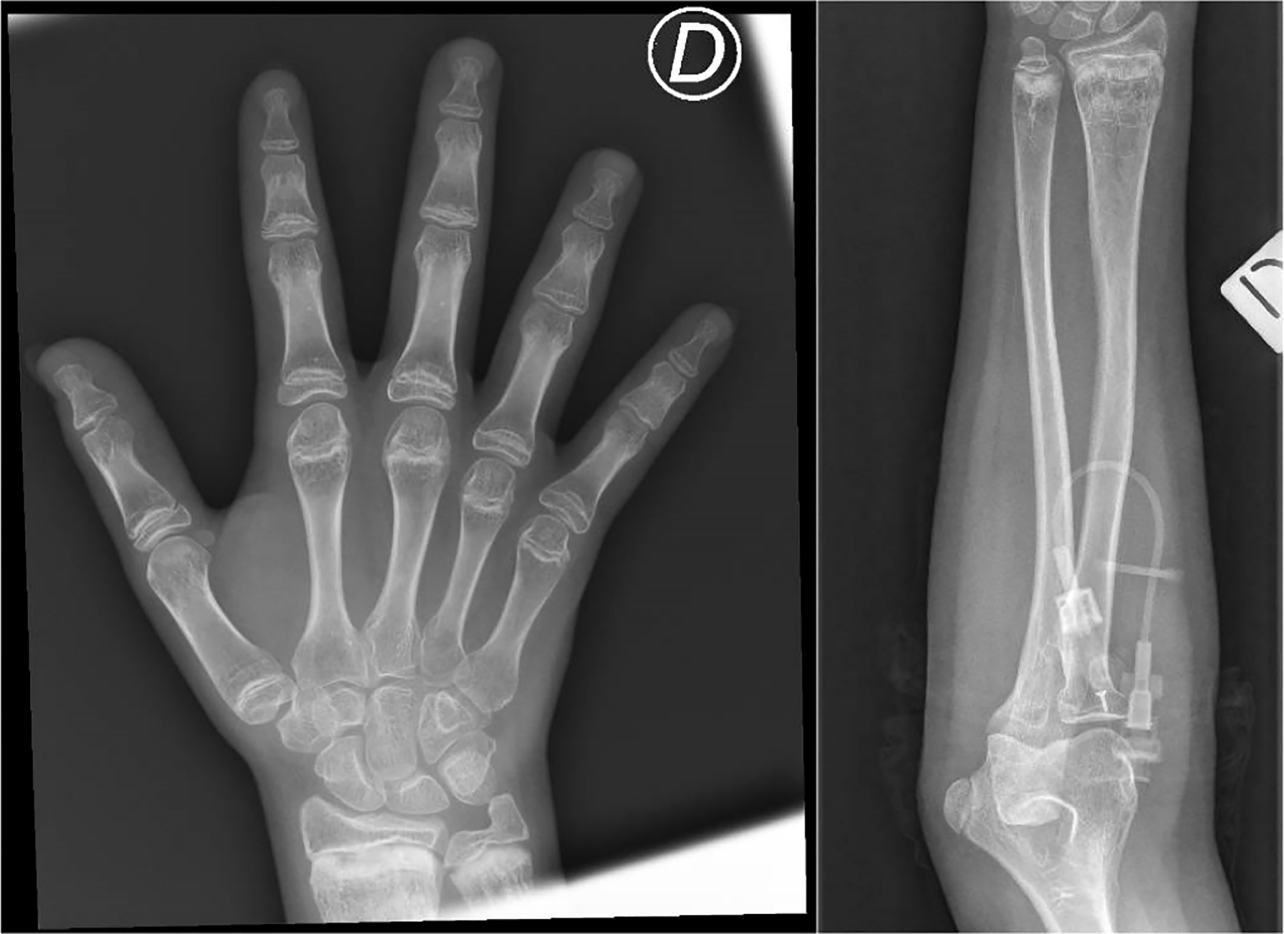
Metaphyseal alterations at the distal radius and ulna, with irregularity of the cartilaginous border and inhomogeneity of the bone structure (areas of marked radiolucency, interspersed among sclerotic striae), and brevity of phalanxes and metacarpal bones. These characteristics are consistent with mild metaphyseal dysplasia in cartilage-hair hypoplasia. D = right.

Over the years, the patient developed progressive lymphopenia with severe B-cell lymphopenia, absence of memory B cells, symptomatic hypogammaglobulinemia with recurrent respiratory tract infections, and bronchiectasis, treated with subcutaneous immune globulins and azithromycin. Chronic inflammatory skin disease and recurrent non-infectious diarrhea also appeared. Progressive growth delay associated with growth hormone deficiency poorly responding to replacement therapy also developed, which became particularly evident in peri-pubertal years (final height at 15 years of age: 142.5 cm, corresponding to −2.9 standard deviation from average). A total body radiological skeletal survey then revealed metaphyseal dysplasia (**Figure 1**). At age 15 years, she developed severe headache, neck stiffness, photophobia, wide-based gait, and microzoopsia. She was diagnosed with cryptococcal meningoencephalitis and successfully treated with daily liposomal amphotericin B for 4 weeks and flucytosine for 2 weeks, followed by 10 weeks of fluconazole at a therapeutic dosage, later continued at a prophylactic dosage.

Sirolimus was stopped and the patient rapidly became anemic (Hb nadir at 6 g/dl, 6 weeks after therapy interruption); marrow evaluation again showed features of erythropoietic failure, with severely impaired erythroid colony formation and increased DNTs up to 3%. Once infection resolved, we estimated that the benefit of sirolimus on erythropoiesis outbalanced the mild immunosuppressive effect, and thus, we restarted treatment that led to resolution of anemia within 3 weeks. The patient had no neurological sequelae or cryptococcosis relapses following this episode.

Whole-genome sequencing (WGS) identified two compound heterozygous mutations in the gene encoding the long non-coding RNA *RMRP* (ENST00000363046.1): a maternally inherited single-nucleotide rare variant, n.67G>A (rs1245480029), and a novel paternally inherited one-nucleotide insertion, n.64_65insA. Both variants map to a proximal highly conserved region of the gene, where several other variants associated with CHH–anauxetic dysplasia spectrum disorders are localized. For this reason, the two variants detected in the proband were considered pathogenic, and a diagnosis of CHH was made.

The progressively severe immune deficiency along with the cryptococcal meningoencephalitis—that in these patients, despite chronic antifungal treatment, has a high risk of lethal recurrence—strongly supported the indication to HSCT. In the absence of a matched family or unrelated donor, she received at 15 years of age a haploidentical alpha-beta-T-depleted HSCT from her heterozygous, clinically, and immunologically healthy father. The conditioning regimen included thiotepa, treosulfan, and fludarabine. No toxic events or recurrence of cryptococcal meningoencephalitis occurred during the early period after HSCT; sustained neutrophil and platelet engraftment occurred within 16 days post-infusion. At the time of writing, the patient is 2 months post-transplant and shows normal blood counts with no signs of graft-versus-host disease and full donor chimerism. She will receive fluconazole prophylaxis until immunological reconstitution is complete.

## Discussion

In this brief report, we describe a case of CHH presenting with a clinical phenotype of ALPS and erythroid marrow failure, in whom sirolimus corrected severe anemia and normalized DNT cells; the patient was genetically diagnosed with the use of WGS. We believe that this description is interesting for three main reasons. First, it shows that the immunological profile of CHH may mimic that found in ALPS and ALPS-related disorders. Second, it shows the novel finding of the efficacy of sirolimus in treating the associated severe anemia. Third and last, it highlights the crucial role of WGS in achieving a definitive diagnosis in unresolved complex cases.

As for the immunological phenotype, CHH has been associated with the propensity for recurrent and atypical infections and for the tendency toward poly-organ autoimmunity and immune dysregulation similar to that observed in ALPS and in other PIRDs like autoimmune regulator (*AIRE*) deficiency (4, 12). Deficient RMRP function determines an aberrant thymic architecture and thymic hypoplasia, which ultimately impairs T-lymphocyte proliferation and directly increases T-lymphocyte apoptosis (13). Recent evidence (3) links RMRP deficiency to the pathologically upregulated PI3K-Akt signaling pathway, which is involved in multiple PIRDs (6, 14) and is effectively inhibited by sirolimus (15). Although we did not test PI3K functional status in our patient, it can be speculated that PI3K inhibition favored the control of our patient’s immunodysregulatory features, as indicated by the resolution of splenomegaly, a decrease in B220-positive DN T-cell count, and DAT negativization under sirolimus treatment. Consistent with what is observed in other PIRDs (16), unfortunately, mTOR inhibition did not prevent the progression of the immunodeficiency that ultimately generated the indication to HSCT.

The overlap between the clinical and immunologic features of CHH and ALPS has not been previously recognized; thus, this report effectively expands the spectrum of the possible phenotypes associated with *RMRP* mutations. In this respect, we acknowledge two limitations of this case study: since we did not perform a targeted sequencing of the FAS gene in sorted DNT cells, we cannot exclude the fact that the immunodysregulatory features shown by the patient were driven by a somatic FAS mutation, a mechanism involved in approximately 20%–30% of ALPS cases with no detectable germline mutation (17); in addition, no functional validation of pathogenicity was available for the two *RMRP* mutations found in the patient, which are formally considered to be variants of unknown significance. Nonetheless, the profound immunodeficiency and metaphyseal dysplasia displayed by the patient are not consistent with a diagnosis of ALPS, and perfectly fit with CHH; moreover, the familial segregation and the fact that both variants fall in mutational hot spots for the gene likely support the attribution of pathogenicity.

To the best of our knowledge, the use of sirolimus has never been previously reported in CHH. Sirolimus proved very effective in correcting erythropoietic failure in our patient. This effect is clearly demonstrated by the improvement of all tested parameters, including *in vitro* erythroid progenitor growth on drug initiation, their worsening on sirolimus withdrawal, and their return to normal when the drug was reprised. There is no obvious explanation for this. In *Drosophila* models of human ribosomopathies, sirolimus proved to stimulate autophagy (i.e., the protective mechanism that maintains homeostasis by removing superfluous and dysfunctional cellular components), thus reducing the ribosomopathy-induced proteotoxic stress and the apoptotic rate (18). In addition, sirolimus increases proliferation of immature erythroblasts in mouse models of other forms of ineffective erythropoiesis, acting through metabolic reprogramming, decreased oxidative stress, and autophagy induction (19, 20). Overall, these findings may lead us to speculate that sirolimus acted as an erythropoietic enhancer. Interestingly, due to its erythroid modulating properties, sirolimus is currently being tested in a clinical trial on transfusion-dependent beta-thalassemia patients (THALA-RAP, NCT04247750). In addition, it cannot be excluded that at least part of the erythropoietic failure displayed by the patient was a consequence of ongoing immune dysregulation. Indeed, the biological and clinical overlap between marrow failure syndromes and primary immune deficiencies is increasingly recognized (21): in this light, sirolimus may have acted primarily through immune modulation, as already reported in cases of autoimmune pure red cell aplasia (22). It may be argued that sirolimus might have possibly worsened the immunodeficiency profile of our patient, paving the way for cryptococcal opportunistic infection. On the other hand, sirolimus has been repeatedly shown to be safe and not to increase the rate of severe infections in both PIRD and non-PIRD patients (i.e., those suffering from complicated vascular anomalies) (16, 23). Moreover, the progressive nature of T-lymphocyte deficiency observed in our patient is inherent to CHH immunobiology and is consistent with a previously described case series where no immunosuppressants were reportedly used.

Multiple NGS platforms are now available, and the discussion concerning their optimal application to clinical diagnostics is ongoing. Sequencing panels of disease-specific genes have insofar been the favored approach, due to reduced costs, low turnaround time, and low rate of unspecific and incidental findings. On the other hand, this approach offers the highest diagnostic yield only in cases with unambiguous clinical finding where low genetic heterogeneity is expected. Indeed, when a patient presents with atypical or complex combinations of clinical abnormalities, untargeted analytical platforms such as WES or WGS (24), as used in this report, may well be considered. In fact, WGS is the most comprehensive solution in this scenario, due to its theoretical capability to identify nearly all forms of genetic variation (25). In particular, it remains the most reliable method to explore the non-protein-coding region of the genome and identify intergenic and intronic pathogenic variants, or mutations in RNA-coding genes, as also shown in our report. Also, obtaining a comprehensive data set with WGS allows for increased diagnostic yields in case of future reanalysis, if new clinical features arise in a specific patient, or if new disease–gene relationships are discovered. Our report thus supports the expanding role of WGS as an important asset to the clinical care for individuals with rare disorders.

## Conclusions

We report on a CHH patient whose diagnosis was made through WGS, in whom sirolimus was able to steadily correct anemia and part of immunodysregulation, thus suggesting a potential implementation of the therapeutic armamentarium in CHH.

## Data Availability Statement

The original contributions presented in the study are included in the article/supplementary material. Further inquiries can be directed to the corresponding author.

## Author Contributions

GDB collected the clinical data and drafted the first version of the manuscript. CD, MM, IC, AG, MF, UR, and NDI wrote sections of the manuscript. ML performed the hematopoietic precursor growth assays. IC, AG, MR, AC, and SG performed genetic sequencing, bio-informatic analysis of the genetic data, and its interpretation. All authors critically revised the manuscript, read, and approved the submitted version.

## Conflict of Interest

The authors declare that the research was conducted in the absence of any commercial or financial relationships that could be construed as a potential conflict of interest.

## Publisher’s Note

All claims expressed in this article are solely those of the authors and do not necessarily represent those of their affiliated organizations, or those of the publisher, the editors and the reviewers. Any product that may be evaluated in this article, or claim that may be made by its manufacturer, is not guaranteed or endorsed by the publisher.
